# The influence of clinical experience on reliable evaluation of pharyngeal and laryngeal lesions: comparison of high-definition laryngoscopy using narrow band imaging with fibre-optic laryngoscopy

**DOI:** 10.1017/S0022215123001846

**Published:** 2024-04

**Authors:** Constanze Scholman, Manon A Zwakenberg, Jan Wedman, Jan E Wachters, Gyorgy B Halmos, Bernard F A M van der Laan, Boudewijn E C Plaat

**Affiliations:** 1 Department of Otorhinolaryngology – Head and Neck Surgery, University of Groningen, University Medical Center Groningen, Groningen, the Netherlands; 2 Department of Otorhinolaryngology – Head and Neck Surgery, Haaglanden Medical Center, The Hague, the Netherlands

**Keywords:** Laryngoscopy, reproducibility of results, laryngoscopes, narrow band imaging, referral and consultation

## Abstract

**Background:**

Fibre-optic laryngoscopy is still widely used in daily clinical practice; however, high-definition laryngoscopy using narrow band imaging could be more reliable in characterising pharyngeal and laryngeal lesions.

**Methods:**

Endoscopic videos were assessed in a tertiary referral hospital by 12 observers with different levels of clinical experience. Thirty pairs of high-definition laryngoscopy with narrow band imaging and fibre-optic laryngoscopy videos were judged twice, with an interval of two to four weeks, in a random order. Inter- and intra-observer reliability, sensitivity and specificity were calculated in terms of detecting a malignant lesion and a specific histological entity, for beginners, trained observers and experts.

**Results:**

Using high-definition laryngoscopy with narrow band imaging, inter-observer reliability for detecting malignant lesions increased from moderate to substantial in trained observers and experts (high-definition laryngoscopy with narrow band imaging κ = 0.66 and κ = 0.77 *vs* fibre-optic laryngoscopy κ = 0.51 and κ = 0.56, for trained observers and experts respectively) and sensitivity increased by 16 per cent.

**Conclusion:**

Inter-observer reliability increased with the level of clinical experience, especially when using high-definition laryngoscopy with narrow band imaging.

## Introduction

Worldwide, otolaryngologists use endoscopic assessment to diagnose pharyngeal and laryngeal lesions on a daily basis.^1^ In the out-patient setting, the fibre-optic laryngoscope is still commonly used for laryngeal examination.^2^ In the last few decades, medical imaging technology has improved immensely, and nowadays high-definition laryngoscopy can be combined with narrow band imaging.^3^ It enables the laryngologist to visualise superficial mucosal vascular patterns specifically.^4^

Narrow band imaging uses blue and green wavelengths that correspond to the haemoglobin peak absorption spectrum, resulting in a high-contrast visualisation of blood vessels. The superficial mucosa capillaries absorb blue light and subepithelial blood vessels absorb green light.^4^ (Pre)malignancies are characterised by aberrant vessel patterns. Previous studies showed high accuracy to detect a lesion, and possibly a better clinical outcome by adding narrow band imaging to high-definition laryngoscopy.^5^ The combination of high-definition laryngoscopy with narrow band imaging improved clinical differentiation between malignant and benign lesions, compared to high-definition laryngoscopy alone.^6^

Besides accuracy, which indicates the correctness of an observation, precision is a key element for the evaluation of a diagnostic test.^7^ Precision shows the agreement between observers (inter-observer reliability). Furthermore, intra-observer reliability indicates whether the results given by the same observer are consistent.^8^ Although earlier studies report the benefits of narrow band imaging when compared to high-definition laryngoscopy, high-definition laryngoscopy with or without narrow band imaging has not yet been implemented by many otolaryngologists, and fibre-optic laryngoscopy remains widely used.^9,10^ In addition, no previous studies have compared inter- and intra-observer reliability between the most advanced and available imaging technique of high-definition laryngoscopy with narrow band imaging versus the most used method of fibre-optic laryngoscopy. Furthermore, clinical reasoning of the physician plays a significant role in diagnostic decision-making. Experts use analytical reasoning, whereas beginners are influenced by non-analytical reasoning.^11^

The primary aim of this study was to compare the inter- and intra-observer reliability of high-definition laryngoscopy with narrow band imaging versus standard fibre-optic laryngoscopy, for characterising pharyngeal and laryngeal lesions. The secondary aim was to assess the influence of clinical experience on precision, sensitivity and specificity in detecting pharyngeal and laryngeal lesions.

## Materials and methods

This retrospective study was approved by the institutional review board of the University Medical Center Groningen, and it was judged that this study (number: 201800677) did not fall under the scope of the Dutch Medical Research Involving Human Subjects Act (the Netherlands ‘WMO’ law). This study was performed according to the Standards for Reporting of Diagnostic Accuracy Studies (‘STARD’) 2015 guidelines.

In total, data from 30 videolaryngoscopies (both high-definition laryngoscopy with narrow band imaging and fibre-optic laryngoscopy) were used: 24 videolaryngoscopies extracted from our archived database and 6 videos of healthy volunteers with a normal larynx. Patient images were routinely collected between June 2014 and October 2017. Inclusion criteria were the availability of high-definition laryngoscopy with narrow band imaging and fibre-optic laryngoscopy videos of the same lesion, and no treatment between both recordings. All detected lesions were histopathologically confirmed; no biopsies were taken from a normal larynx. The high-definition laryngoscopy with narrow band imaging and the fibre-optic laryngoscopy videos were recorded within a median interval of 1.5 days (standard deviation = 15.4, range = 0–78 days).

Videos were recorded with a flexible video rhinolaryngoscope (Olympus ENF VH; Olympus Medical Systems, Tokyo, Japan) connected to a high-definition monitor for high-definition laryngoscopy with narrow band imaging, and with a flexible fibre-optic rhinolaryngoscope (Olympus ENF GP; Olympus Medical Systems) attached to a Matrix E camera processor (Xion, Berlin, Germany) for fibre-optic laryngoscopy. Videos, patient data and histopathological results were documented from the electronic patient records.

Videos were edited using Adobe Premiere Pro video editing software (Adobe, San Jose, California, USA) to compose fragments with a maximum duration of 20 seconds, in which images as close as possible to the lesion were used, aiming for near-contact endoscopy. The high-definition laryngoscopy with narrow band imaging videos consisted of high-definition laryngoscopy and narrow band imaging fragments alternately. Both the high-definition laryngoscopy with narrow band imaging videos and the fibre-optic laryngoscopy videos showed an overview of the lesion location.

A questionnaire was composed in Microsoft Access 2010 (Microsoft, Redmond, Washington, USA) with all videos and patient characteristics (gender, age, intoxications, brief summary of patient medical history and earlier cancer treatment), to simulate daily clinical practice. For each observer, a new questionnaire was created, and the videos presented in a random order.

Videos were presented once or, on request of the observer, twice at the most, using a high-definition screen (Samsung model code: UE50ES6100, software version: T-MST10PDEUC-1032.0 BT-G, Samsung, Seoul, South Korea). After classifying the lesion as malignant or benign, observers had to choose a diagnosis from a presented table (Table 1). The table included a variety of diagnoses, and not all diagnoses were included. The researcher notified the observer when half of the videos had been completed and before the last 10 videos. All observers judged the same lesions again after two to four weeks. The same videos were presented in a random order to reduce confounding effects such as recognition and fatigue of observers. Observers consisted of four ENT consultants (three head and neck oncology surgeons and one laryngologist with at least 12 years’ experience in interpreting endoscopic images), six ENT residents and two ENT researchers (with 1.5 and 2 years’ experience in interpreting endoscopic images).

**Table 1.** Possible diagnosis choices and lesion locations in the videos

Parameter	Frequency of videos (*n* (%))
Diagnosis	
– Normal	7 (23.3)
– Cyst	0 (0)
– Metaplasia, hyperplasia	1 (3.3)
– Scar tissue, granulation	1 (3.3)
– Hyperkeratosis, parakeratosis	3 (10)
– Lymphoid tissue	2 (6.7)
– Inflammation	2 (6.7)
– Radiotherapy sequelae	1 (3.3)
– Severe dysplasia/carcinoma in situ	0 (0)
– Squamous cell carcinoma	13 (43.3)
Lesion location	
– Glottis	9 (30)
– Supraglottis	4 (13.3)
– Hypopharynx	3 (10)
– Oropharynx	5 (16.7)
– Glottis & supraglottis	2 (6.7)
– Normal pharynx & larynx	7 (23.3)

For statistical analysis, observers were divided into three groups of four observers in terms of their level of clinical experience: experts (four ENT consultants), trained observers (ENT residents in at least their fourth year of training) and beginners (two ENT residents, both in their first year of training, and two researchers). Statistical analysis was performed using SPSS version 22.0 (IBM, Armonk, New York, USA). Generalised Fleiss kappa values (κ-values) for multiple raters were used to calculate inter-observer reliability. Cohen's kappa values were implemented to determine intra-observer reliability. The description of kappa values by Landis and Koch was applied for interpretation of kappa values.^12^ The chi-square test was used to analyse differences in sensitivity and specificity.

## Results

In total, 30 per cent of lesions were located in the larynx, 16.7 per cent in the oropharynx and 10 per cent in the hypopharynx (Table 1). Seven videos showed a normal larynx (23.3 per cent). The ratio of men to women was 19 to 11. In total, 22 of the 30 patients had no previous ENT medical history (73.3 per cent). Three patients underwent transoral laser surgery, two patients had radiotherapy and one patient had chemoradiation previously. Furthermore, two patients had undergone prior neck dissection.

### Inter-observer reliability

**Figure 1.**
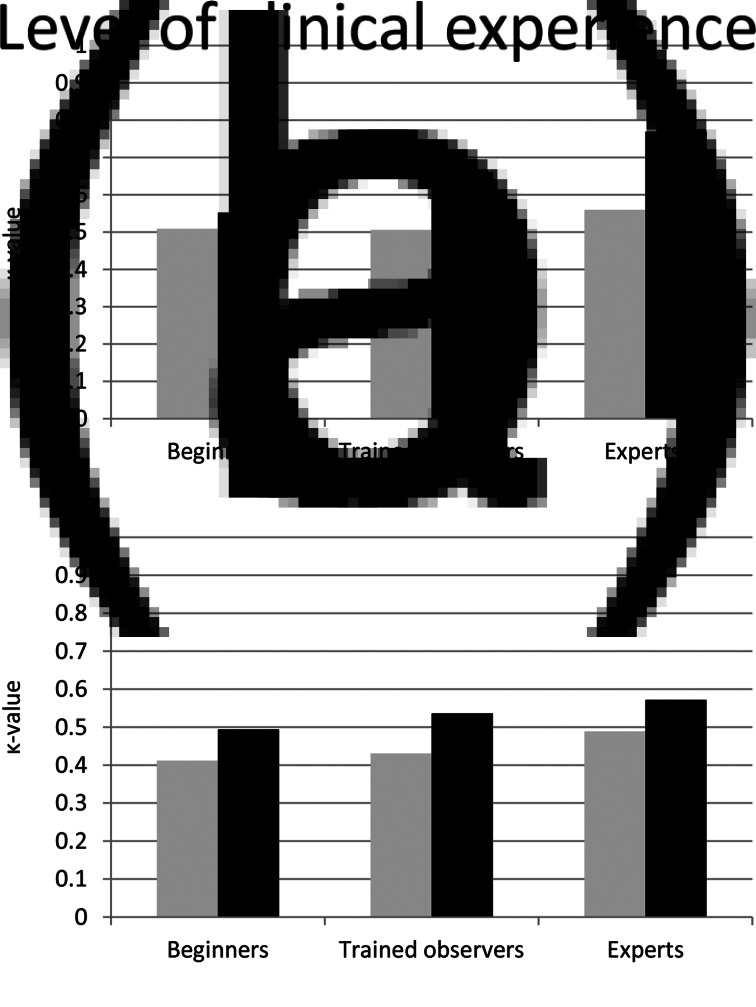
Inter-observer reliability for detecting (a) a malignant lesion and (b) a specific histological entity, for high-definition laryngoscopy with narrow band imaging (black) and fibre-optic laryngoscopy (grey).

As demonstrated in Figure 1, high-definition laryngoscopy with narrow band imaging achieved a higher inter-observer reliability for detecting a malignant lesion, in all three observer groups. Experts showed the highest reliability having a substantial agreement (κ = 0.77, Table 2). In the trained observers and experts group, agreement increased from moderate (using fibre-optic laryngoscopy) to substantial (using high-definition laryngoscopy with narrow band imaging) for recognising a malignant lesion. For identifying a specific histological entity, inter-observer reliability was moderate in all three groups for both laryngoscopes, but the κ-value for beginners, trained observers and experts was higher for high-definition laryngoscopy with narrow band imaging (high-definition laryngoscopy κ = 0.49, κ = 0.54 and κ = 0.57, respectively) compared to fibre-optic laryngoscopy (κ = 0.41, κ = 0.43 and κ = 0.49, respectively).

**Table 2.** Inter- and intra-observer reliability for detecting malignant lesions and specific histological entities, for beginners, trained observers and experts

Parameter	Detection of malignant lesion		Detection of specific histological entity	
	HDL-NBI	Fibre-optic laryngoscopy	HDL-NBI	Fibre-optic laryngoscopy
Inter-observer reliability				
– Beginners	0.55	0.51	0.49	0.41
– Trained observers	0.66	0.51	0.54	0.43
– Experts	0.77	0.56	0.57	0.49
Intra-observer reliability				
– Beginners	0.59	0.63	0.55	0.60
– Trained observers	0.83	0.62	0.69	0.63
– Experts	0.70	0.75	0.68	0.66

Data represent κ-values. HDL-NBI = high-definition laryngoscopy with narrow band imaging

### Intra-observer reliability

**Figure 2.**
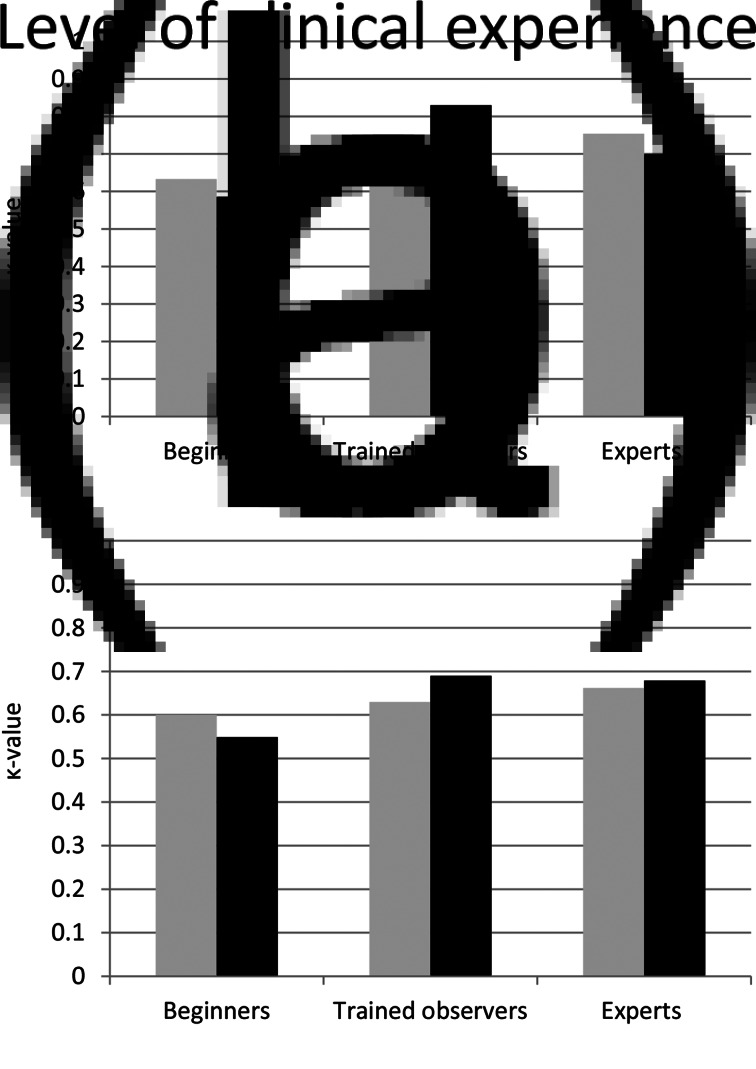
Intra-observer reliability for detecting (a) a malignant lesion and (b) a specific histological entity, for high-definition laryngoscopy with narrow band imaging (black) and fibre-optic laryngoscopy (grey).

For trained observers, the κ-value was markedly higher for detecting a malignant lesion using high-definition laryngoscopy with narrow band imaging, showing almost perfect agreement, compared to fibre-optic laryngoscopy (high-definition laryngoscopy with narrow band imaging κ = 0.83 *vs* fibre-optic laryngoscopy κ = 0.62; Table 2). Beginners and experts had a higher κ-value using fibre-optic laryngoscopy to distinguish a malignant from a benign lesion (high-definition laryngoscopy with narrow band imaging κ = 0.59 and κ = 0.70 *vs* fibre-optic laryngoscopy κ = 0.63 and κ = 0.75, for beginners and experts respectively). For identifying a specific histological entity, trained observers and experts showed substantial agreement for both laryngoscopes, whereas beginners demonstrated moderate agreement (Figure 2).

### Sensitivity and specificity

Overall sensitivity for detecting a malignant lesion was highest using high-definition laryngoscopy with narrow band imaging (high-definition laryngoscopy with narrow band imaging 85.0 per cent *vs* fibre-optic laryngoscopy 77.8 per cent, *p* = 0.01; Table 3). Within the different observer groups, sensitivity was higher for high-definition laryngoscopy with narrow band imaging, but was only significant for experts when detecting a malignant lesion (high-definition laryngoscopy with narrow band imaging 96.2 per cent *vs* fibre-optic laryngoscopy 78.8 per cent, *p* = 0.01). All observer groups showed significantly higher sensitivity for high-definition laryngoscopy with narrow band imaging compared to fibre-optic laryngoscopy when identifying a specific histological entity (*p* < 0.05). Experts had the highest sensitivity for both laryngoscopes when detecting a malignant lesion and a specific histological entity (high-definition laryngoscopy with narrow band imaging 96.2 per cent and 76.1 per cent *vs* fibre-optic laryngoscopy 78.8 per cent and 60.9 per cent, for detecting a malignant lesion and specific histological entity respectively).

**Table 3.** Sensitivity and specificity for detecting malignant lesions and specific histological entities, for high-definition laryngoscopy with narrow band imaging and fibre-optic laryngoscopy

Parameter	Detection of malignant lesion				Detection of specific histological entity			
	HDL-NBI	Fibre-optic laryngoscopy	χ^2^ value	*p*-value	HDL-NBI	Fibre-optic laryngoscopy	χ^2^ value	*p*-value
Sensitivity								
– Beginners	76.9 (40 of 52)	73.1 (38 of 52)	0.21	0.65	62 (57 of 92)	43.5 (40 of 92)	6.3	0.01*
– Trained observers	88.5 (46 of 52)	76.9 (40 of 52)	2.42	0.12	71.7 (66 of 92)	55.4 (51 of 92)	5.3	0.02*
– Experts	96.2 (50 of 52)	78.8 (41 of 52)	7.12	0.01*	76.1 (70 of 92)	60.9 (56 of 92)	4.9	0.03*
– Overall sensitivity	85.0 (136 of 156)	77.8 (119 of 156)	6.20	0.01*	69.9 (193 of 276)	53.3 (147 of 276)	16.21	<0.001*
Specificity								
– Beginners	86.8 (59 of 68)	73.5 (50 of 68)	3.74	0.05	75 (21 of 28)	82.1 (23 of 28)	0.04	0.52
– Trained observers	91.2 (62 of 68)	86.8 (59 of 68)	0.67	0.41	75 (21 of 28)	96.4 (27 of 28)	5.25	0.02*
– Experts	86.8 (59 of 68)	89.7 (61 of 68)	0.28	0.6	78.6 (22 of 28)	82.1 (23 of 28)	0.11	0.74
– Overall specificity	88.2 (180 of 204)	83.3 (170 of 204)	2.01	0.16	76.2 (64 of 84)	86.9 (73 of 84)	3.20	0.07

Data represent percentages (numbers) of videos, unless indicated otherwise. *Indicates significant difference. HDL-NBI = high-definition laryngoscopy with narrow band imaging

Specificity for detecting a malignant lesion was not significantly different for any of the three observer groups regarding the laryngoscope (high-definition laryngoscopy with narrow band imaging 86.8 per cent, 91.2 per cent and 86.8 per cent *vs* fibre-optic laryngoscopy 73.5 per cent, 86.8 per cent and 89.7 per cent, for beginners, trained observers and experts, respectively; Table 3). For recognising a specific histological entity, trained observers showed a higher specificity using fibre-optic laryngoscopy compared to high-definition laryngoscopy with narrow band imaging (high-definition laryngoscopy with narrow band imaging 75 per cent *vs* fibre-optic laryngoscopy 96.4 per cent, *p* = 0.02).

## Discussion

To our knowledge, this is the first study to compare precision, diagnostic sensitivity and specificity between high-definition laryngoscopy with narrow band imaging versus fibre-optic laryngoscopy, for detecting pharyngeal and laryngeal lesions, in relation to an observers’ level of clinical experience.

This study showed that high-definition laryngoscopy with narrow band imaging had higher inter-observer reliability for detecting a malignant lesion and a specific histological entity compared to fibre-optic laryngoscopy. By using high-definition laryngoscopy with narrow band imaging instead of fibre-optic laryngoscopy, the inter-observer reliability for detecting a malignant lesion increased from moderate to substantial in trained observers and experts (high-definition laryngoscopy with narrow band imaging κ = 0.66 and κ = 0.77 *vs* fibre-optic laryngoscopy κ = 0.51 and κ = 0.56, for trained observers and experts respectively). Moreover, high-definition laryngoscopy with narrow band imaging demonstrated the highest intra-observer reliability for identifying a malignant lesion, having almost perfect agreement in the trained observers group (κ = 0.83). Furthermore, the sensitivity of high-definition laryngoscopy with narrow band imaging was superior to that for fibre-optic laryngoscopy in recognising a specific histological entity and detecting a malignant lesion. High-definition laryngoscopy with narrow band imaging showed a higher inter-observer reliability for detecting a malignant pharyngeal and laryngeal lesion and a specific histological entity compared to fibre-optic laryngoscopy, independently of observers’ clinical experience.

An earlier study showed a fair agreement (κ = 0.40) for diagnosing malignant lesions of the upper aerodigestive tract using narrow band imaging, whereas the current study showed substantial (trained observers and experts) and moderate (beginners) agreement using high-definition laryngoscopy with narrow band imaging.^5^ The higher inter-observer reliability in our study could be explained by an increased learning curve of the observers, who became more trained in narrow band imaging after it was introduced to our department in 2014.^13^

Earlier studies did not compare high-definition laryngoscopy plus narrow band imaging with fibre-optic laryngoscopy, but instead investigated the addition of narrow band imaging to high-definition laryngoscopy. Davaris *et al*.^14^ showed an almost perfect agreement (κ = 0.85) and Nogués-Sabaté *et al*.^15^ showed substantial agreement (κ = 0.63) for detecting malignant and benign laryngeal lesions using narrow band imaging. In both studies, agreement increased using high-definition laryngoscopy with narrow band imaging compared to high-definition laryngoscopy alone, showing the importance of narrow band imaging in clinical practice. However, in both studies, images were used instead of videos. We chose to use videos, as this resembles daily clinical practice.

In our study, we can conclude that high-definition laryngoscopy with narrow band imaging had the highest inter-observer reliability and was superior to fibre-optic laryngoscopy. High-definition endoscopy with or without narrow band imaging, or comparable optical filtering techniques, are not the current standard of care in many countries, and fibre-optic laryngoscopy is still widely used in daily clinical practice. Our comparison of standard fibre-optic laryngoscopy with advanced imaging techniques demonstrated the increase in inter-observer reliability of endoscopic diagnosis associated with the use of high-definition narrow band imaging endoscopy.

The current study showed an increase in intra-observer reliability, improving from substantial agreement using fibre-optic laryngoscopy to almost perfect agreement using high-definition laryngoscopy with narrow band imaging, for trained observers detecting a malignant lesion (κ = 0.83). Davaris *et al*.^14^ presented similar results using narrow band imaging for detecting a laryngeal malignancy, but only patients with laryngeal anomalies were included, and the analysed near-contact method does not resemble our daily clinical practice. Nogués-Sabaté *et al*.^15^ showed only substantial agreement using narrow band imaging for detecting a malignant head and neck lesion. Both of these studies were performed using still images and without additional patient data. This could explain the lower intra-observer reliability of Nogués-Sabaté *et al*.^15^ This limits comparability with our study, in which we opted for a method resembling daily clinical practice, using videos showing an overview of the lesion with the presentation of additional patient data.

Surprisingly, in our study, beginners and experts had a slightly higher κ-value using fibre-optic laryngoscopy compared to high-definition laryngoscopy with narrow band imaging for distinguishing a malignant from a benign lesion. Furthermore, there was no difference in intra-observer reliability for identifying the specific histological entity. This could be explained by a lower image quality of fibre-optic laryngoscopy.^9^ Therefore, there might have been a higher consistency in giving the same diagnosis during both assessments for lesions recorded by fibre-optic laryngoscopy, leading to a higher intra-observer reliability, but with a possible wrong diagnosis. High precision with low accuracy means that many observers are in agreement regarding a wrong diagnosis. Moreover, the visualisation of the vascular pattern using high-definition laryngoscopy with narrow band imaging could possibly lead to higher variability of diagnoses.

Besides precision, we assessed sensitivity and specificity, to further evaluate the performance of both laryngoscopes. Sensitivity of detecting a malignant lesion was highest using high-definition laryngoscopy with narrow band imaging, for all observer groups, and increased significantly within experts (high-definition laryngoscopy with narrow band imaging 96.2 per cent *vs* fibre-optic laryngoscopy 78.8 per cent, *p* = 0.01). We found comparable sensitivities in our earlier study, which showed sensitivities of 91.7 per cent for high-definition laryngoscopy and 79.8 per cent for fibre-optic laryngoscopy for detecting malignant lesions.^9^ Other studies presented a higher sensitivity of narrow band imaging compared to white-light imaging alone.^6,16^ Our findings confirm that high-definition laryngoscopy with narrow band imaging has a higher sensitivity than fibre-optic laryngoscopy for detecting a malignant lesion and a specific histological entity.

The specificity for detecting a malignant lesion was not significantly different between the laryngoscopes, which is comparable to previous results. Specificity was 79.6 per cent for high-definition laryngoscopy and 80.3 per cent for fibre-optic laryngoscopy.^9^ Interestingly, this study showed a lower specificity of high-definition laryngoscopy with narrow band imaging than for fibre-optic laryngoscopy for trained observers identifying a specific histological entity (high-definition laryngoscopy with narrow band imaging 75 per cent *vs* fibre-optic laryngoscopy 96.4 per cent, *p* = 0.02). This could be explained by overdiagnosis when using high-definition laryngoscopy with narrow band imaging because of better visualisation of the vascular pattern.

Many otolaryngologists are still using standard fibre-optic laryngoscopy despite the availability of high-definition laryngoscopy with additional chromoendoscopy. For the first time, we compared the old but widely used endoscopic technique, namely fibre-optic laryngoscopy, with the next step in endoscopic diagnostic procedures, namely high-definition laryngoscopy with the addition of narrow band imaging. Our study utilised a combined analysis of precision (inter- and intra-observer reliability) and accuracy (sensitivity and specificity), and correlated these findings with the level of clinical experience. This study resembled daily clinical practice in the outpatient setting, as the videos were evaluated in combination with the presentation of patient history. The study design was similar to that of our earlier study on high-definition videoendoscopy without narrow band imaging.

We did not include other common benign diagnoses such as vocal fold polyps. The videos were edited into fragments of a maximum duration of 20 seconds. These choices were made to attain a limited time frame for observers to judge videos, in order to minimise the effects of fatigue on observers’ performance. Therefore, we are not able to draw conclusions with regard to other benign lesions. Furthermore, we did not compare high-definition laryngoscopy or fibre-optic laryngoscopy to narrow band imaging only, so we cannot draw conclusions on the effects of narrow band imaging only.

- Fibre-optic laryngoscopy is still widely used; however, high-definition laryngoscopy using narrow band imaging could be more reliable in characterising pharyngeal and laryngeal lesions
- Endoscopic videos were assessed twice, with an interval of two to four weeks, by 12 observers with different levels of ENT clinical experience
- Using high-definition laryngoscopy with narrow band imaging, inter-observer reliability for detecting malignant lesions increased from moderate to substantial in trained observers and experts
- In addition, sensitivity of high-definition laryngoscopy with narrow band imaging increased by 16 per cent
- Inter-observer reliability increased with level of clinical experience, especially when using high-definition laryngoscopy with narrow band imaging

Numerous laryngoscopies are performed in ENT departments all over the world on a daily basis, underlining the importance of precise and accurate laryngoscopy for detecting and diagnosing pharyngeal and laryngeal lesions. This study showed an increase in inter-observer reliability with an observers’ level of clinical experience and when using high-definition laryngoscopy with narrow band imaging. According to our results, the application of high-definition laryngoscopy with narrow band imaging could lead to an earlier diagnosis and therefore an improved prognosis. Furthermore, overall sensitivity for detecting a malignant lesion and a specific histological entity was higher using high-definition laryngoscopy with narrow band imaging, emphasising the superior performance of detecting lesions using high-definition laryngoscopy with narrow band imaging instead of fibre-optic laryngoscopy.

## Conclusion

The inter-observer reliability increased from moderate to substantial in trained observers and experts when detecting a malignant lesion using high-definition laryngoscopy with narrow band imaging instead of the widely used fibre-optic laryngoscopy technique. Moreover, the sensitivity of high-definition laryngoscopy with narrow band imaging was superior to that of fibre-optic laryngoscopy when recognising a specific histological entity. The use of high-definition laryngoscopy with narrow band imaging not only leads to a more accurate detection, but also to a more precise diagnosis of pharyngeal and laryngeal lesions.
